# Antimicrobial Activity of Oregano Essential Oil Incorporated in Sodium Alginate Edible Films: Control of *Listeria monocytogenes* and Spoilage in Ham Slices Treated with High Pressure Processing

**DOI:** 10.3390/ma12223726

**Published:** 2019-11-12

**Authors:** Foteini Pavli, Anthoula A. Argyri, Panagiotis Skandamis, George-John Nychas, Chrysoula Tassou, Nikos Chorianopoulos

**Affiliations:** 1 Institute of Technology of Agricultural Products, Hellenic Agricultural Organization-DEMETER, Lycovrissi, 14123 Attica, Greece; photpavli@gmail.com (F.P.); anthi.argyri@gmail.com (A.A.A.); ctassou@nagref.gr (C.T.); 2 Laboratory of Microbiology and Biotechnology of Foods, Department of Food Science and Human Nutrition, Agricultural University of Athens, 11855 Athens, Greece; gjn@aua.gr; 3 Laboratory of Food Quality Control and Hygiene, Department of Food Science and Human Nutrition, Agricultural University of Athens, 11855 Athens, Greece; pskan@aua.gr

**Keywords:** *Listeria monocytogenes*, spoilage, Ready-To-Eat meat, antimicrobial packaging, antimicrobial compounds, non-thermal processing, Infrared spectroscopy

## Abstract

The aim of the study was to evaluate the efficacy of oregano essential oil (OEO) incorporated in Na-alginate edible films when applied to sliced ham inoculated with a cocktail of *Listeria monocytogenes* strains, with or without pretreatment by high pressure processing (HPP). Microbiological, physicochemical and sensory analyses (in *Listeria*-free slices) were performed, while, the presence/absence and the relative abundance of each *Listeria* strain, was monitored by pulsed field gel electrophoresis (PFGE). The OEO incorporation in the films, caused approximately 1.5 log reduction in *Listeria* population at 8 and 12 °C at the end of the storage period, and almost 2.5 log reduction at 4 °C. The HPP treatment caused 1 log reduction to the initial *Listeria* population, while levels kept on decreasing throughout the storage for all the tested temperatures. The pH of the samples was higher in the cases where HPP was involved, and the samples were evaluated as less spoiled. Furthermore, the presence of OEO in the films resulted in color differences compared to the control samples, whilst the aroma of these samples was improved. In conclusion, the combined application of HPP and OEO edible films on the slices, led to a significant reduction or absence of the pathogen.

## 1. Introduction

*Listeria monocytogenes* is a widespread food contaminant of major safety concern especially in ready-to-eat (RTE) food products. In 2017, 2480 cases of listeriosis were reported in Europe, with 1.8% overall occurrence of *Listeria monocytogenes* in RTE meat products [1], although other pathogenic bacteria such as *Salmonella*, Shiga-toxin producing *E. coli* and *Staphylococcus aureus*, could also be found in such products. *Listeria monocytogenes* is a psychrotrophic bacterium and has the ability to proliferate at various temperatures and environmental conditions [2]. This pathogen can be spread within food processing plants via raw materials, equipment and human activities, and is able to adhere to many food contact surfaces. Furthermore, the prevalence of *Listeria monocytogenes* in RTE products which are preserved under refrigeration such as deli products, is of major concern due to the fact that are generally consumed without further processing [3].

RTE meat products that have undergone thermal treatment during processing are usually deemed as safe and free of pathogens [4]. Among RTE meat products, sliced ham remains on demand, due to consumers’ modern lifestyle. Although ham is a thermally-processed product, other activities such as slicing and handling considerably increase the likelihood of post-processing contamination. Consequently, ensuring safety during the entire shelf-life, without adding or increasing food additives or preservatives is a challenge for food manufacturers worldwide [5]. On the other hand, particularly for sliced ham, the quality can be easily compromised, due to the development of off-aromas, rancidity, and issues related to the appearance of the product [6]. Such issues have encouraged research in new technologies for RTE products in order to inhibit microbial growth, while ensuring quality, freshness and nutritional value [7]. 

Several methods have been investigated to extend shelf-life or decontaminate food products. Non-thermal alternative processing technologies have been widely accepted throughout the food industry [8]. Especially for sliced cooked meat products a promising technology is high pressure processing (HPP). HPP is a processing technique, in which microorganism inactivation occurs with the use of high pressure; usually above 300 MPa, while the processing temperature does not increase beyond 40 °C [8,9]. HPP causes destruction of microbial vegetative cells and enzyme inactivation, without adverse effects on the sensory characteristics of the product [10]. However, the efficacy of the treatment depends on the applied pressure, the time of exposure and the treatment’s temperature [9,10]. Details regarding the technology of HPP for meat products have been reported previously [10,11,12,13]. The HPP treatment can be satisfactorily combined with new packaging systems or natural antimicrobial compounds in the sense of a “hurdle concept”, where all the factors can act synergistically, to reach the objective of minimal processing, without compromising the products’ safety [4]. 

Active packaging is a system where the product, the package and the package environment interact with each other [14]. Active packaging technologies aim to extend the shelf-life and enhance the safety by retarding or even inhibiting the spoilage or the growth of pathogenic microorganisms, and also maintain or improve the properties of the packaged food [15,16]. The mechanism of this type of packaging is based on the incorporation of active compounds into the packaging material to absorb substances from the food or its environment, or to release agents from the packaging to the food [14,17]. Antimicrobial active packaging is a type of active packaging, which has received a lot of attention by both researchers and industries, with numerous commercial applications so far. Antimicrobial substances may be released through evaporation or migrated into food through diffusion and partitioning, directly with application on the food surface or indirectly with their incorporation into carrier materials such as coatings or edible films [18]. Other antimicrobial effects can also be provided with this technology through oxygen scavenging systems, moisture absorbing systems, carbon dioxide generation, ethanol generation, and humidity buffering [14]. Active packaging and antimicrobial packaging technologies have been reviewed elsewhere in detail [14,18,19,20,21,22,23,24,25]. 

Natural or chemical antimicrobial compounds may be used in antimicrobial active packaging. Due to the fact that the use of chemical substances raises safety concerns to the consumers and efforts have been made to eliminate their use, the application of natural antimicrobial compounds is of great interest [26]. Antimicrobial compounds such as bacteriocins, enzymes, ethanol or essential oils, have been studied thoroughly to determine their effectiveness against spoilage and pathogenic microorganisms in food matrices [27,28,29,30,31,32,33]. Essential oils (EO) are aromatic natural substances extracted by steam distillation from aromatic plants, herbs and spices and most of them are classified as generally recognised as safe (GRAS). Many studies are available regarding the *in vitro* antimicrobial properties of certain EOs, with quite low values of minimal inhibitory concentrations (MIC) against pathogens, including *Listeria monocytogenes*. Reports have shown that higher concentrations of EOs are required for food applications compared to laboratory media, due to their interactions with food components [34]. Numerous studies have confirmed that the EO of oregano (*Origanum vulgare*) is very effective against food spoilage and pathogenic microorganisms [35,36] and it has been used as antimicrobial agent in meat and meat products to control *Listeria monocytogenes* [34,37,38,39]. The chemical composition of oregano EO includes a mixture of *p*-cymene, *α*-terpinene, thymol and carvacrol, with the latter two components being the most important due to their effects on bacterial membranes [40]. Additionally, it has been reported that these compounds have the ability to protect against oxidation processes [41]. 

It has to be noted that the use of EOs as food preservatives is still limited because of their intense flavour [16,26,42]. An alternative methodology to minimize the sensory effects can be the incorporation of EOs into polymer matrices such as edible films/coatings, leading to the reduction of the organoleptic impact of the substances, while their diffusion to the product can be controlled [42,43]. As an edible film can be defined a layer of material that is applied to the surface of a food product and can normally be consumed with it. Edible films can be particularly useful as carriers of essential oils, due to their ability to maintain high concentrations of these substances on the food surface [44]. The incorporation of essential oils in edible films has a great impact on the control of bacterial growth during the storage of meat and meat products, since contamination usually occurs on the surface of such products [45]. Sodium alginate is a polysaccharide that has received a lot of attention as a film matrix due to its biocompatibility and low toxicity. This material is usually obtained from seaweeds and has the ability to form a strong film that presents better performances when compared to other materials regarding water permeability and mechanical properties [46]. It is important to be mentioned that alginate has been applied to meat and meat products, since it delays dehydration and eliminates the effects of lipid oxidation [47]. 

Since sublethally injured pathogens could be more susceptible to antimicrobial compounds such as EOs, the combined application of HPP and other preservation technologies could theoretically increase pathogen inactivation, while at the same time the use of relatively low pressure levels enhances the preservation of the quality characteristics of the product. The objective of the present study was to evaluate the effectiveness of different treatments and their combination to control *Listeria monocytogenes* during storage of vacuum-packed sliced ham at different temperatures (4, 8 and 12 °C). The treatments used were i) application of HPP, ii) antimicrobial packaging using Na-alginate edible films with oregano essential oil, and iii) a combination of these two technologies. The effect of the different treatments on the shelf-life of the product was also assessed.

## 2. Materials and Methods 

### 2.1. Bacterial Strains and Cocktail Preparation

Three strains of *Listeria monocytogenes*, kindly provided by the laboratory of Microbiology and Biotechnology of Foods of Agricultural University of Athens (Food Microbiology Culture Collection-FMCC), were used in the present study. The strains, namely FMCC-B-129, FMCC-B-131 and FMCC-B-133, were originated from Greek industries. More specifically, the three strains were isolated from RTE frozen minced meat meal, conveyor belt of RTE frozen food and from soft cheese, respectively. The pure cultures were stored in −80 °C in brain heart infusion broth (BHI, LabM, Lancashire, UK) supplemented with 20% (*v*/*v*) glycerol. The strains were subcultured twice in BHI broth for 24 h and 18 h at 37 °C, before use. Bacterial cells were harvested separately by centrifugation at 10,000 g for 10 min, washed in ¼ strength Ringer’s solution (this step was performed twice), and finally resuspended in 10 mL of the aforementioned solution. The three-strain cocktail was prepared by mixing the three strains in equal volumes. The final mixture was used to inoculate ham slices at an approximate level of 4 log CFU/g. Inoculum size was confirmed by serial dilutions and plating on Palcam Agar base (LabM, Lancashire UK), for *Listeria* spp., incubated at 30 °C for 48 h.

### 2.2. Ham Slices 

Sliced ham in commercial packages was purchased from a local supermarket (Athens, Greece) with dimensions 10 cm × 10 cm, 2 mm thick and 20 g approximate weight per slice. Due to a possible contamination, the first 5 slices coming out from the cutting machine were removed completely, as well as all the slices that were in direct contact with the packaging material provided by the supermarket. Ham slices were inoculated with the *Listeria* cocktail at levels of *ca.* 4 log CFU/g, and were then processed according to each case scenario. Ham slices were also prepared, without the addition of *Listeria monocytogenes* and were used for sensory evaluation. Three storage temperatures were examined in the present study (4, 8 and 12 °C), while two independent experiments were performed with duplicate samples in each experiment. On each independent experiment, ham slices by different manufacturer were used. 

### 2.3. High Pressure Processing (HPP) Treatment

HPP treatment was conducted at the pressure of 500 MPa for 2 min at room temperature (20 °C), when applied according to the case. Both pressure and temperature were monitored and recorded during the process with 1 s intervals, while for the pressurization time reported (2 min) the pressure come-up and release times were excluded. Details regarding the high pressure equipment and operating conditions are available elsewhere [48,49]. 

### 2.4. Preparation and Application of Na-Alginate Edible Films

The preparation of Na-alginate edible films was performed as previously described by Kapetanakou et al. [18]. Briefly, 2 g of Na-alginate (Applichem GmbH, DarmstadtCity, Germany) was added gradually in 100 mL of pre-warmed (65 °C) distilled sterile water and under constant agitation for complete dissolution. A total of 1 mL of glycerol was used as a plasticizer in order to improve the film’s flexibility. After the addition of the glycerol, the forming solution was kept at 4 °C for approximately 30 min to lower the temperature, until the addition of the essential oil. Oregano essential oil (Ecopharm, Hellas) was added under stable agitation at a final volume of 1% (*v*/*v*) in the forming solution, for the preparation of the oregano essential oil-supplemented edible films (OEOS). Na-alginate solution without the addition of oregano essential oil was also prepared for the oregano essential oil-free edible films (OEOF). Films were produced in square Petri-dishes using 20 g of Na-alginate forming solution and were immediately placed in a laminar flow cabinet at room temperature to dry for 12 h. Once the films were dry, aliquots of 20 mL of 2% (*w*/*v*) CaCl_2_ were added in the Petri-dishes for 1 min, so that the films get detached (ca. 0.5 g). After the preparation, the films were stored under refrigeration until their final application (less than 24 h), when needed.

### 2.5. Microbiological Analysis

Microbiological analyses were carried out throughout the storage period in all temperatures tested. Samples of ham slices (10 g) were weighed aseptically, added to sterile ¼ strength Ringer’s solution (90 mL) (LabM, Lancashire, UK), and finally homogenized in a stomacher (Stomacher 400 Circulator, Seward Limited, Norfolk, UK) for 1 min at room temperature. Serial decimal dilutions were prepared in the same diluent and the appropriate dilutions were poured or spread using 1 or 0.1 mL, respectively. The agar media used were the following: de Man-Rogosa-Sharpe (MRS) medium (CM 1153, Oxoid, Hampshire, UK) for lactic acid bacteria (LAB), overlaid with the same medium and incubated at 30 °C for 72 h; M17 Agar (4017192, Biolife, Milano, Italy) for lactococci/streptococci, incubated at 30 °C for 72 h; Plate Count Agar (LAB149, LabM, Lancashire, UK) for total viable counts (TVC), incubated at 30 °C for 48 h; Streptomycin Thallous Acetate-Actidione Agar (STAA, CM0881, supplemented with selective supplement SR0151, Biolife, Milano, Italy) for *Brochothrix thermosphacta*, incubated at 25 °C for 48 h; Rose Bengal Chloramphenicol Agar (LAB036 supplemented with selective supplement X009, LabM, CityLancashire, UK), for yeasts/molds, incubated at 25 °C for 5 days; Violet Red Bile Glucose Agar (CM0485, Oxoid, Hampshire, UK) for *Enterobacteriaceae*, incubated at 37 °C for 24 h; Pseudomonas Agar base (LAB108 supplemented with selective supplement X108, LabM, Lancashire, UK), for *Pseudomonas* spp., incubated at 25 °C for 48 h; as well as Palcam Agar base (LAB148 supplemented with selective supplement X144, LabM, Lancashire, UK), for *Listeria* spp., incubated at 30 °C for 48 h. In cases where the levels of *Listeria* were below the detection limit of the enumeration method, enrichment was followed according to ISO 11290-1:1996/Amd 1:2004 [50], using the following media: Half Fraser broth (Biolife, Milano, Italy) incubated at 30 °C for 24 h, Fraser broth (Biolife, Milano, Italy), incubated at 37 °C for 48 h and Agar Listeria Ottaviani and Agosti (ALOA, Biolife, Milano, Italy), incubated at 37 °C for 24 or 48 h.

### 2.6. Isolation of Listeria Cells and Strain Differentiation 

Ham slices were analyzed at specific time intervals during their storage at the different temperatures and from all the cases tested (with or without HPP treatment, with or without edible films (either OEOF or OEOS). From specific time points, approximately 20% of the colonies were randomly collected from the appropriate dilution on Palcam agar base or ALOA, after the enrichment step. The isolates, after checked for their purity, were stored in −80 °C in BHI broth, supplemented with 20% (*v*/*v*) glycerol. Before further analysis, each isolate was grown twice in BHI broth at 37 °C for 24 h. 

For *Listeria monocytogenes* typing, pulsed field gel electrophoresis (PFGE) was used according to Kagkli et al. [51]. The restriction enzyme ApaI (10U) (New England Biolabs, Ipswich, MA, USA) was used in line with the recommendations from the manufacturer for 18 h. After the digestion step, restriction fragments were separated in 1% PFGE grade agarose gel in 0.5 mM Tris-Borate buffer on a CHEF-DRIII (BIO-RAD, Hercules, CA, USA) equipment with the following running parameters: 6 V cm^−1^, 1 s initial switching time, 40 s final switching time and 18 h total run at 14 °C. The obtained restriction profiles were then compared to the PFGE fingerprints of the inoculated *Listeria* strains. 

### 2.7. pH and Color Determination

The pH value of the samples was recorded with a digital pH meter (HI 2211 pH-ORP Meter, HANNA Instruments, Woonsocket, RI, USA), during storage at different temperatures at different time intervals. After finishing the microbiological analysis, the ham homogenate (stomacher homogenate) was used to measure the pH of the samples. 

The color of the ham samples was evaluated by taking at least five random readings from the surface of the different samples using a Minolta Chroma Meter fitted with CR-300 measuring head (Minolta, Osaka, Japan). Measurements of the instrument were standardized with respect to a white calibration plate, every time before use. The CIE (Commission Internationale de l’Eclairage), L*, a*, b*, colorimetry system was used for color determination with L* representing lightness, a* representing redness and b* representing yellowness. All of the measurements were collected from areas on the ham surface without visual excess fat, and the values were recorded for C* (chroma) calculation using the following equation: C* = (a*^2^ + b*^2^)^1/2^. Values regarding control and OEOF samples (without essential oil), were common with those reported in our previous paper [52], while the results relevant to the essential oil supplemented films—OEOS (which is the purpose of this paper) are reported for first time in the current paper. 

### 2.8. Sensory Evaluation

Sensory evaluation of ham slices (non-pathogen inoculated samples) was performed during storage at all temperatures, according to Gill and Jeremiah [53], while further details regarding the design of the sensory evaluation were previously reported [52]. It has to be noted that, the results regarding sensory evaluation of control and OEOF samples (without essential oil), were common with those reported in our previous paper [52], while the results relevant to essential oil supplemented films—OEOS (which is the purpose of this paper) were reported for first time in the current paper. Briefly, a trained sensory panel of 5 members was used and sensory analysis was performed at the same time intervals with the microbiological sample points. The same 5 trained members were used throughout the sensory evaluation and were always blinded to the tested sample. Sensory evaluation was conducted under artificial light in individual booths in a sensory analysis room allocated in the Institute of Technology of Agricultural Products of Hellenic Agricultural Organization—DEMETER. The selected descriptors were based on the perception of aroma, taste and appearance. Each attribute was scored on a three-point hedonic scale ranging from 1 (fresh) to 3 (unacceptable), since the aim of the sensory evaluation was the detection of changes with regards to aroma, taste and appearance that are related to spoilage. Intermediate sensory qualities were attributed to scores of 1.5, 2 and 2.5, while scores > 2 indicated the end of shelf-life and classified the product as spoiled. A total value was calculated as a mean value of the aroma, taste and appearance, rounded to the closest value. 

### 2.9. FTIR Analysis

The FTIR analysis was performed as previously described by Pavli et al. [54]. Briefly, for the FTIR analysis a ZnSe 45° attenuated total reflectance (ATR) flat plate crystal was used on a Perkin Elmer Frontier FTIR spectrometer equipped with a DLaTGS detector with KBr window (PerkinElmer Inc., Seer Green, UK). The spectrometer collected spectra over the wavenumber range 4000–650 cm^−1^ and was programmed with PerkinElmer Spectrum v10.4.2 software (Seer Green, UK). The FTIR analysis included 10 scans per measurement with a resolution of 4 cm^−1^. FTIR spectra between 4000 and 650 cm^−1^ were used for further analysis.

#### 2.9.1. Data Analysis and Partial Least Squares (PLS) Modeling

The Unscrambler software (version 9.7, CAMO, Oslo, Norway) was used to perform the data analysis. The collected FTIR spectral data were standardized at the beginning using standard normal variate (SNV) transformation. Then, the normalized data were mean centered and subjected to partial least squares (PLS) analysis to examine the relationships between FTIR spectral data and the data from the microbiological analyses. The data were divided in two data sets, one batch of ham samples was used for the training of the model (50%) and the other one for the validation (50%). PLS-R models were built for the quantitative analysis of the LAB, lactococci/streptococci and TVC population, using the FTIR data as input variables and the counts of each microbial group as output variables. 

#### 2.9.2. Evaluation of Model Performance

For the prediction of LAB, lactococci/streptococci and TVC in each sample, the following performance indices were calculated; accuracy (A*_f_*) and bias (B*_f_*) factors [55] together with root mean squared error (RMSE) and the percentage of prediction error (%PE). Prediction errors (PE, in log10) were calculated as PE = O-P, where O is the observed value (log), P is the predicted value (log), while a PE < 0 indicates fail-safe predictions and PE > O indicates fail-dangerous predictions. 

The acceptable prediction zone (APZ) was established as −1.0 log < acceptable PE < 0.5 log, and it was twice as wide in the fail-safe area, since a larger error can be tolerated in the fail-safe area when a model is used to predict food safety [56]. 

The percentage of PE (%PE) falling within the APZ served as an overall assessment of the model’s performance and PE total is the total number of PE in the evaluation. A %PE of > 70% indicated a simulation model that provided acceptable predictions for the validation data set [57].

### 2.10. Statistical Analysis 

All experiments were carried out in duplicate with two independent batches of ham slices each. The results from different treatments were analyzed using multifactor analysis of variance in order to test the effect of independent main factors (treatment, bacterial populations, pH, color, sensory scores) on the dependent variable (*Listeria monocytogenes* counts). Means were compared with Duncan post-hoc tests and differences were considered as significant at a 5% level. The differences regarding the factor “pressure” were evaluated using the student T-test to compare the means of the two different groups (with and without HPP treatment). The statistical analysis was performed with IBM® SPSS® Statistics for Windows software, Version 24.0 (IBM Corp., New York, NY, USA).

## 3. Results

### 3.1. Microbiological Results

**Figure 1:**
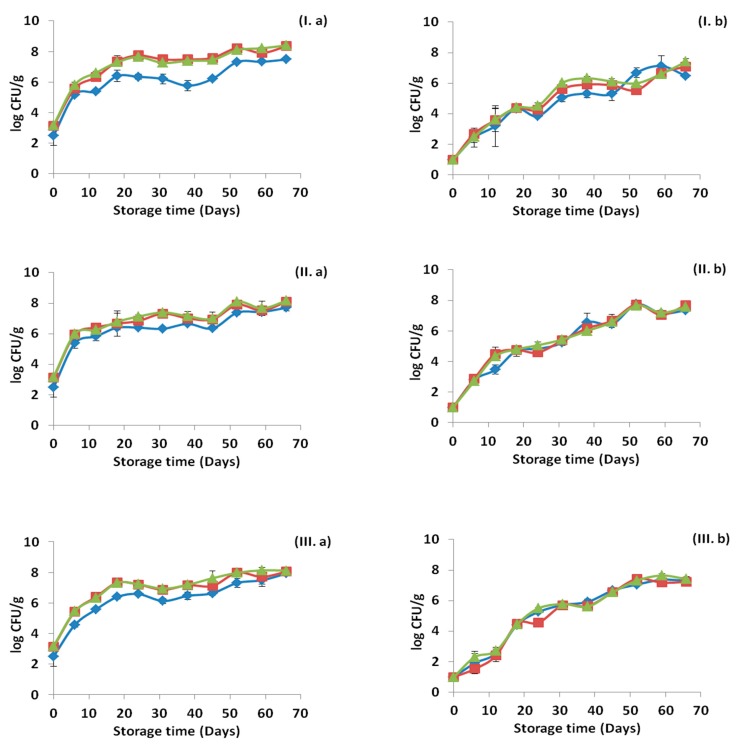
Growth curves of the different bacterial populations in ham stored at 4 °C for the control samples (**I**), samples with edible film free from oregano essential oil- (OEOF) (**II**) and samples with edible film supplemented with oregano essential oil- OEOS (**III**), without (**a**) and after (**b**) the high pressure processing treatment. (♦) Lactic acid bacteria, (■) lactococci/streptococci and (▲) total viable counts. The bars represent the mean values ± standard deviations.

**Figure 2:**
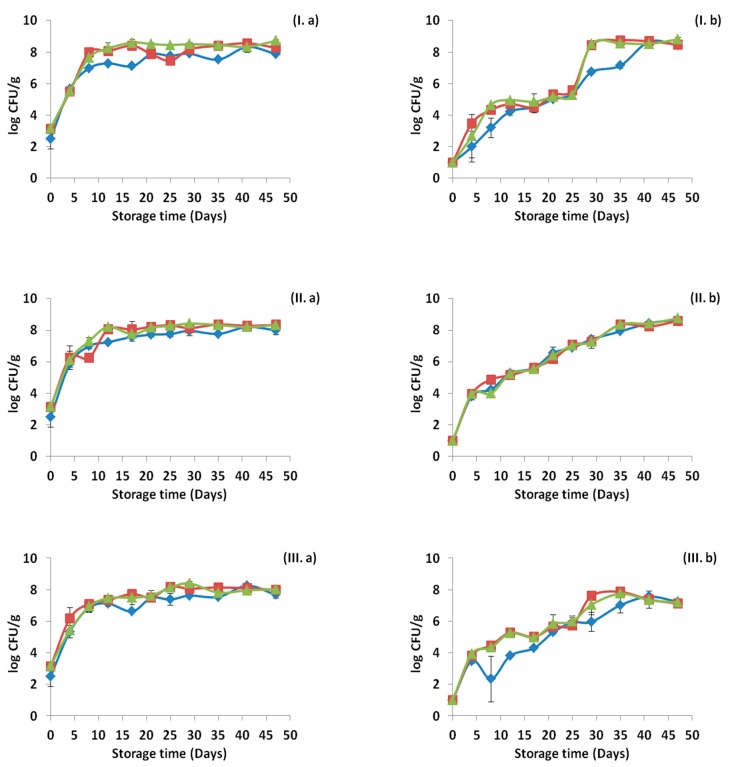
Growth curves of the different bacterial populations in ham stored at 8 °C for the control samples (**I**), samples with edible film free from oregano essential oil-OEOF (**II**) and samples with edible film supplemented with oregano essential oil-OEOS (**III**), without (**a**) and after (**b**) the high pressure processing treatment. (♦) Lactic acid bacteria, (■) lactococci/streptococci and (▲) total viable counts. The bars represent the mean values ± standard deviations.

**Figure 3:**
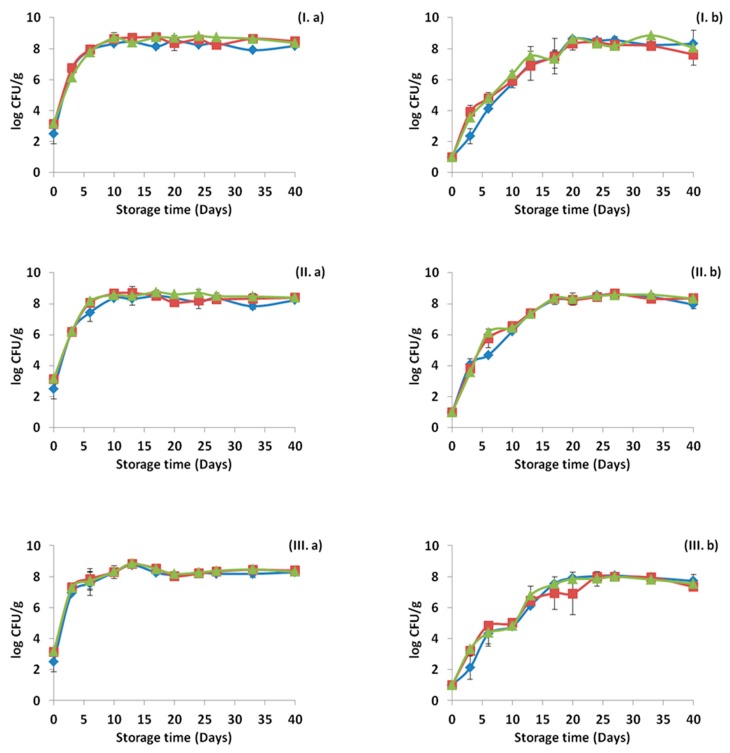
Growth curves of the different bacterial populations in ham stored at 12 °C for the control samples (**I**), samples with edible film free from oregano essential oil-OEOF (**II**) and samples with edible film supplemented with oregano essential oil-OEOS (**III**), without (**a**) and after (**b**) the high pressure processing treatment. (♦) Lactic acid bacteria, (■) lactococci/streptococci and (▲) total viable counts. The bars represent the mean values ± standard deviations.

Microbiological analysis was performed in all samples for each treatment and the results are presented in Figure 1, Figure 2 and Figure 3, for the storage temperature of 4, 8 and 12 °C, respectively. The initial population of ham was 2.51 ± 0.67 for LAB, 3.15 ± 0.11 for lactococci/streptococci and 3.19 ± 0.09 log CFU/g for TVC. When pressure was applied the initial population of ham decreased to 1 log CFU/g for LAB, lactococci/streptococci and TVC. As shown in Figure 1, Figure 2 and Figure 3, all bacterial populations increased in counts during the storage in all temperatures. However, in the cases of HPP treatment, all microbial groups reached their maximum population in later time points depending on the storage temperature. In general, differences were observed between the counts of the bacterial populations in some of the cases, with the lowest counts exhibited by the LAB, while counts of lactococci/streptococci and TVC were very similar throughout the storage period. The application of HPP treatment resulted to intense variations in the standard deviations at each time point. Furthermore, slightly lower bacterial counts were observed in the case of OEOS edible films application. LAB, lactococci/streptococci and TVC were affected significantly (*p* < 0.05) by “pressure” and “time” at 4, 8 and 12 °C. The factor “treatment” affected significantly (*p* < 0.05) the LAB and lactococci/streptococci at 4 °C, but not the TVC counts (*p* > 0.05). Differently, all bacterial counts were affected (*p* < 0.05) by “treatment” at storage temperatures of 8 and 12 °C. When OEOS edible film was used, the bacterial counts of all populations were significantly lower (*p* < 0.05), regardless the storage temperature. It has to be noted that in every sampling point, other microbial populations such as *Brochothrix thermosphacta*, *Pseudomonas* spp., *Enterobacteriaceae* and yeasts/molds were always below the detection limit (<1 log CFU/g).

**Figure 4:**
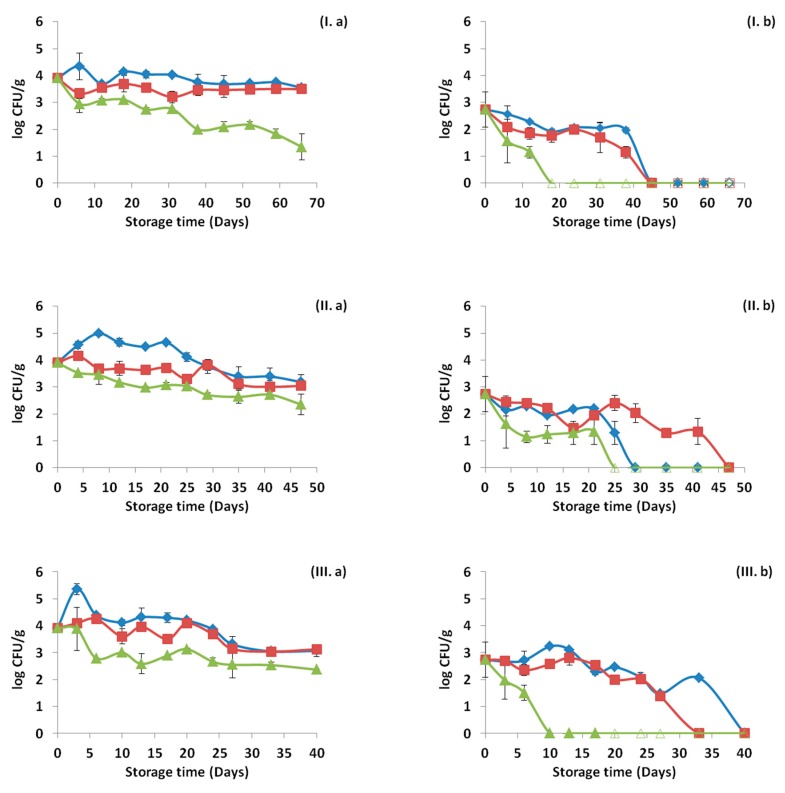
Survival curves of *Listeria monocytogenes* cocktail strains in ham stored at 4 °C (**I**), 8 °C (**II**) and 12 °C (**III**), without (**a**) and after (**b**) high pressure processing treatment. (♦) Control samples, (■) samples with edible film free from oregano essential oil-OEOF and (▲) samples with edible film supplemented with oregano essential oil-OEOS. Open symbols (◊, □, Δ), indicate absence of *Listeria monocytogenes* after application of the enrichment method. The bars represent the mean values ± standard deviations.

Growth curves of *Listeria* are presented in Figure 4, for every treatment and storage temperature. The initial inoculum of *Listeria* was 3.91 ± 0.11 log CFU/g in the ham slices, with the application of HPP treatment reducing it to 2.74 ± 0.65 log CFU/g. For the cases without HPP treatment, *Listeria* mean counts were higher (*p* < 0.05) compared to the samples with OEOS edible films for the three temperatures. *Listeria* growth was not observed in any case throughout the storage time. Additionally, the application of HPP treatment, led to a decrease in *Listeria* counts in undetectable levels, before or at the end of the shelf-life, while the combination of HPP and OEOS edible films led to such decrease far before the end of the shelf-life at all storage temperatures. All the factors; “pressure”, “treatment” and “time” were found to affect significantly (*p* < 0.05) the counts of *Listeria* at all storage temperatures. Regarding the factor “treatment” lower mean values were observed for the case of OEOS edible films application. However, slightly higher mean counts of *Listeria* were observed in the control case, compared to the case of OEOF edible films application. 

### 3.2. pH and Color Measurements

**Figure 5:**
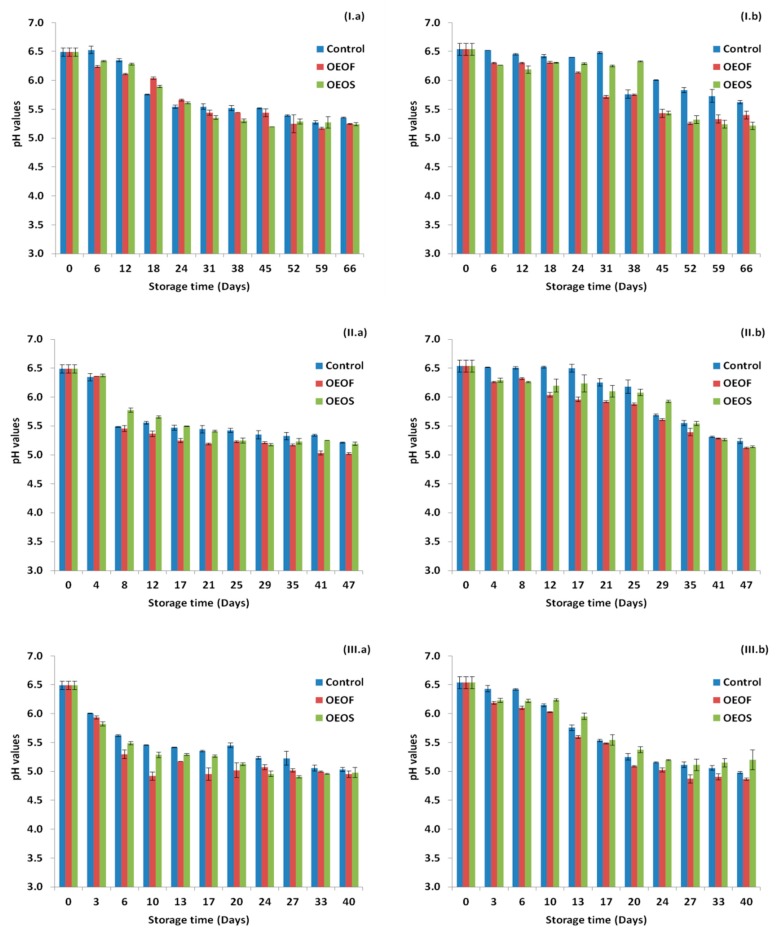
Changes in pH values for ham slices without (**a**) and after (**b**) high pressure processing treatment, during storage at 4 °C (**I**), 8 °C (**II**) and 12 °C (**III**). Values regarding control and OEOF samples (without essential oil), are common with those reported in our previous study [52].

**Figure 6:**
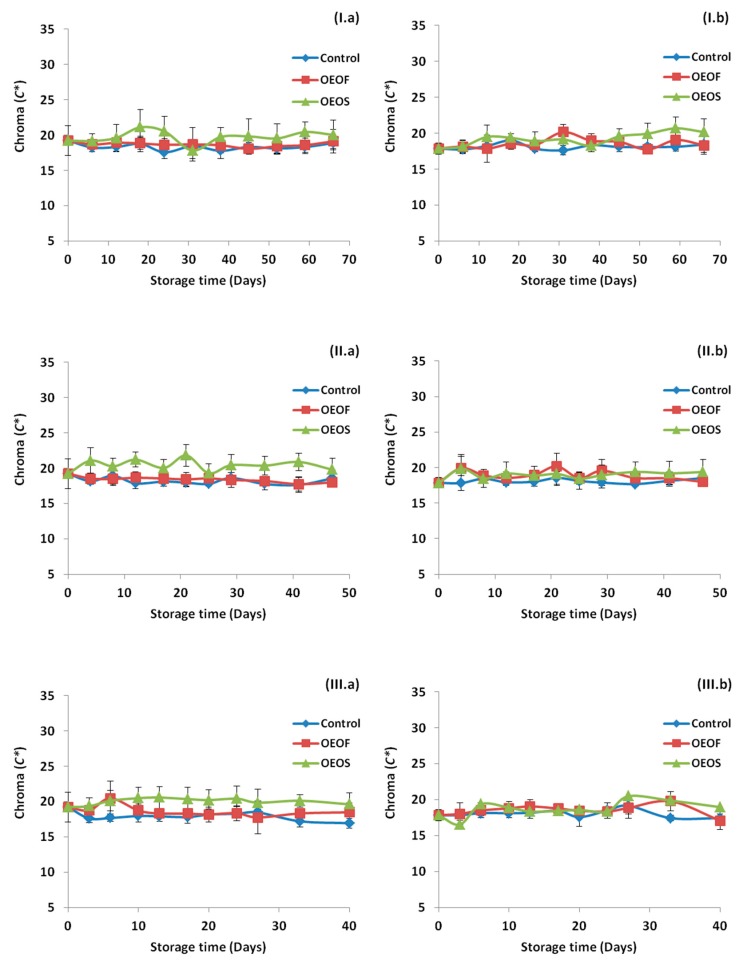
Changes in C* values for ham slices without (**a**) and after (**b**) high pressure processing treatment, during storage at 4 °C (**I**), 8 °C (**II**) and 12 °C (**III**). Values regarding control and OEOF samples (without essential oil), are common with those reported in our previous study [52].

Figure 5 demonstrates the changes in the pH during storage at the three storage temperatures and for all the cases tested. The pH values for all the cases were decreased by time, and the decrease was more intense in the cases where HPP treatment was not applied. In addition, storage temperature affected the final values of the pH, with the lowest ones to be observed at the storage temperature of 12 °C (abuse temperature). The pH was affected by the factors “pressure”, “treatment” and “time” (*p* < 0.05) in all temperatures tested (4, 8 and 12 °C). In Figure 6, the C* values are presented for each case and for the three storage temperatures. The application of OEOS edible films affected the mean values of C* (higher values) and such differences were observed in all temperatures. Differently, the factors “pressure” and “time” did not affect significantly (*p* > 0.05) the C* values at any temperature. 

### 3.3. Sensory Assessment

**Figure 7:**
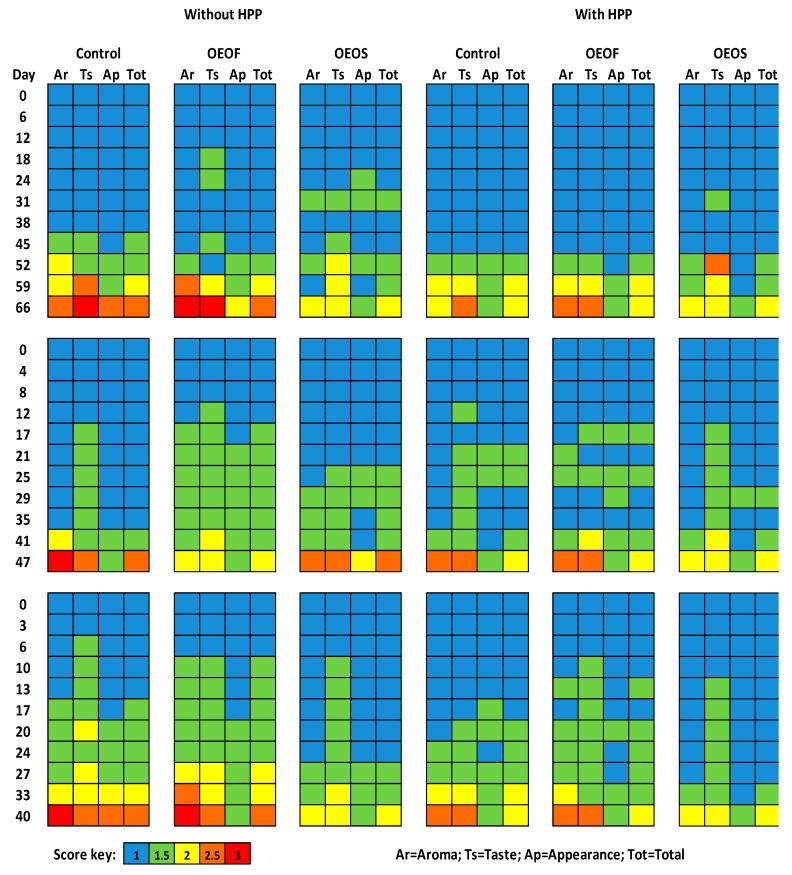
Sensory scores for ham slices without and after the high pressure processing treatment, during storage at 4 (upper line), 8 (middle line) and 12 °C (bottom line). The “total” value represents the mean value of aroma, taste and appearance of each sample rounded to the closest value. Scores regarding control and OEOF samples (without essential oil), are common with those reported in our previous study [52].

The results of the sensory evaluation of the different treatments and cases are presented in Figure 7. The application of HPP affected significantly the sensory characteristics of the ham slices in all storage temperatures and lower scores (more fresh/less spoiled) were given by the panelists compared to the untreated samples for the same time points. The presence of edible films supplemented with oregano essential oil (OEOS), improved the aroma scores given by the panelists in most of the cases. In addition, the appearance was evaluated better (lower values) in the HPP treated samples compared to the non-treated. As “shelf-life” was established the period of time when the ham slices showed acceptable sensory characteristics with regard to aroma, taste and appearance, whereas total scores > 2 specified the end of the shelf-life (Figure 7).

### 3.4. Monitoring Survival and Strain Differentiation of Listeria Monocytogenes

**Figure 8:**
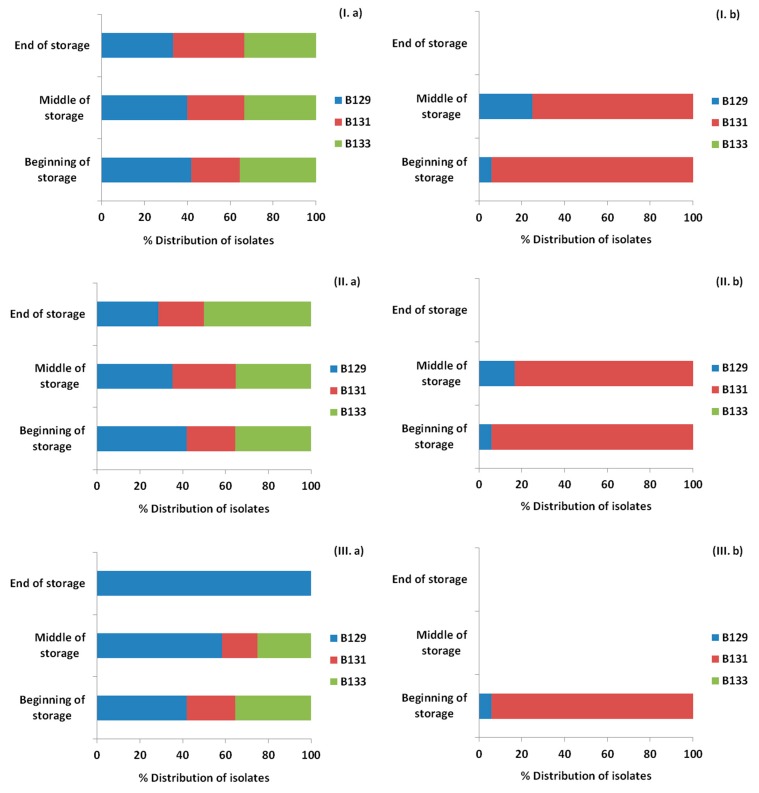
Distribution (%) of *Listeria monocytogenes* strains recovered from ham slices at three time points (beginning, middle, end) during storage at 4 °C based on the pulsed field gel electrophoresis (PFGE) profiles, for control samples (**I**), samples with edible film free from oregano essential oil—OEOF (**II**), samples with edible film supplemented with oregano essential oil—OEOS (**III**), without (**a**) or after (**b**) high pressure processing treatment.

**Figure 9:**
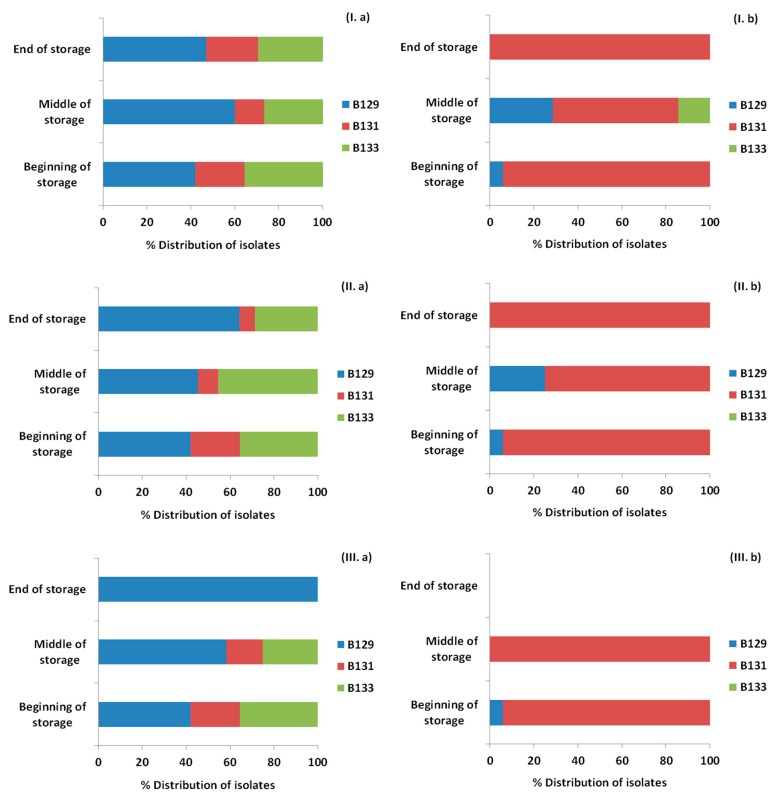
Distribution (%) of *Listeria monocytogenes* strains recovered from ham slices at three time points (beginning, middle, end) during storage at 8 °C based on the pulsed field gel electrophoresis (PFGE) profiles, for control samples (**I**), samples with edible film free from oregano essential oil—OEOF (**II**), samples with edible film supplemented with oregano essential oil—OEOS (**III**), without (**a**) or after (**b**) high pressure processing treatment.

**Figure 10:**
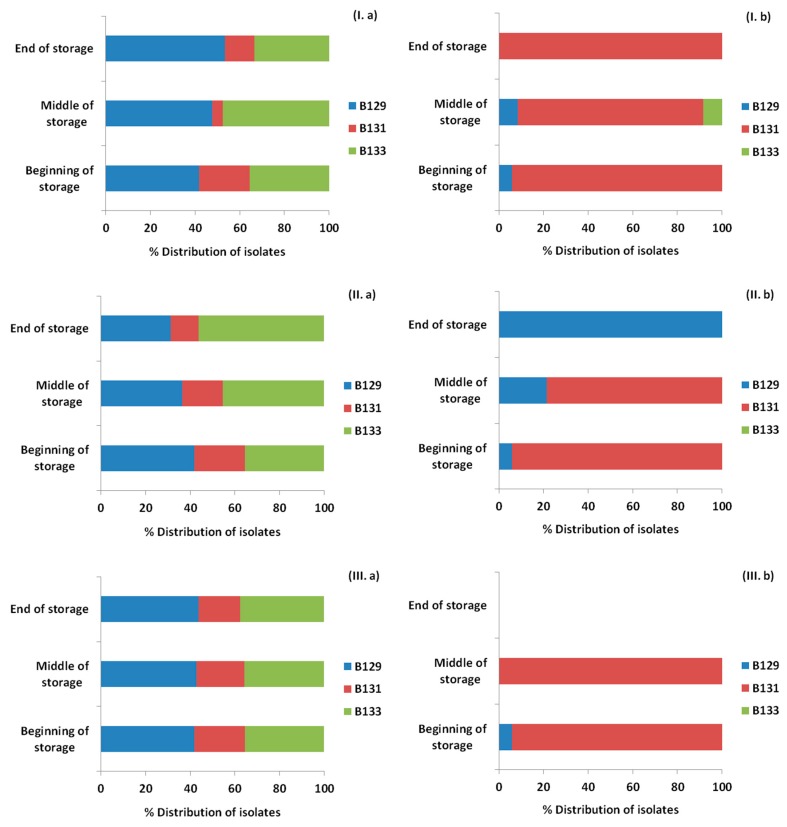
Distribution (%) of *Listeria monocytogenes* strains recovered from ham slices at three time points (beginning, middle, end) during storage at 12 °C based on the pulsed field gel electrophoresis (PFGE) profiles, for control samples (**I**), samples with edible film free from oregano essential oil—OEOF (**II**), samples with edible film supplemented with oregano essential oil—OEOS (**III**), without (**a**) or after (**b**) high pressure processing treatment.

As showed previously, Figure 4 represents the *Listeria* counts for the ham slices for each treatment and storage temperature. Isolates of *Listeria* were recovered from petri dishes of Palcam agar base from the highest dilution or after the enrichment step for each time point and treatment and the presence or absence, as well as the percentage of each of the inoculated strains was confirmed with PFGE. The results are presented in Figure 8, Figure 9 and Figure 10 for the storage temperature of 4, 8 and 12 °C, respectively. 

The initial distribution of the inoculated strains was 41.9% for B129, 22.6% for B131 and 35.5% for B133. The application of OEOS films on the ham slices affected the percentages of recovery of the strains, with the strain B129 being the only one surviving at 4 and 8 °C, while at 12 °C for the same treatment all the strains were detected. In general, it has to be noted that the results obtained from the control and OEOF samples were very similar for the samples without HPP treatment. 

In the case of the HPP application, the initial distribution of the strains was totally different. Specifically, exactly after the HPP treatment the percentages of recovery in the ham slices were 5.9% for B129, 94.1% for B131, while B133 was not detected at all in the beginning of storage period in any case. The only case when the strain B133 was detected after HPP treatment, was in control samples and in the middle of the storage period at 8 and 12 °C. From the results, it is proven that the strain that managed to be recovered widely after the HPP application was B131. *Listeria monocytogenes* strains were not detected in the cases of the combined application of HPP and OEOS edible films, at 4 °C in the middle and end of storage period, with the only exception being the case of 8 and 12 °C, when the strain B131 was recovered after enrichment in the middle of storage, but not in the end. 

### 3.5. FTIR Spectroscopy

**Figure 11:**
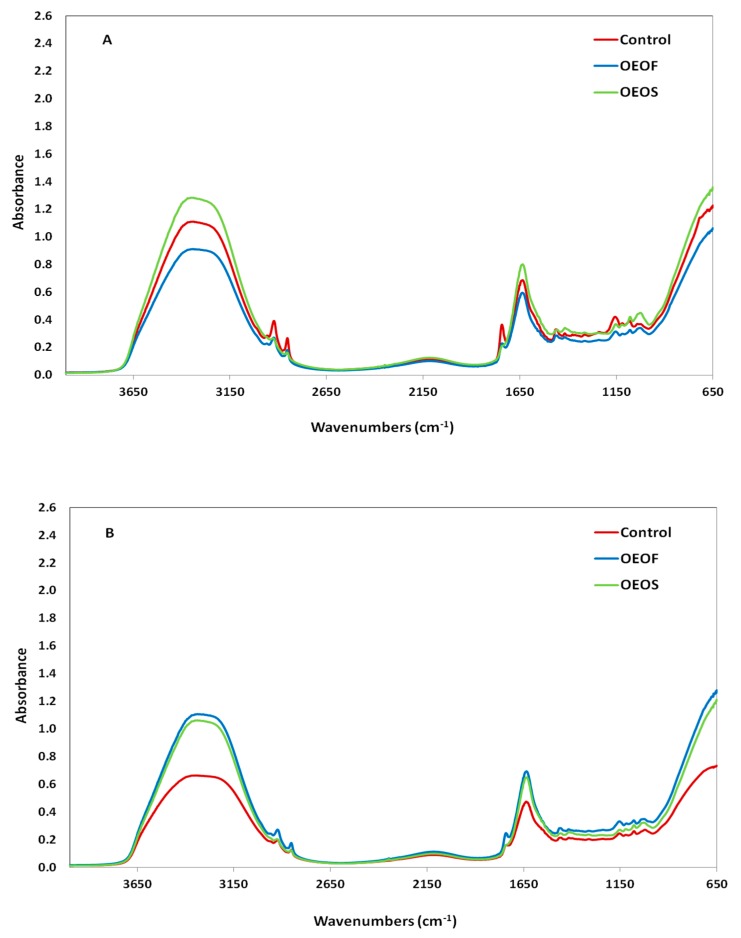
Typical raw FTIR spectra in the range of 4000–650 cm^−1^, at the end of the shelf-life at 12 °C, without (**A**) or after (**B**) high pressure processing treatment for control samples ( **–** ), samples with oregano essential oil-free edible films—OEOF ( **–** ), and samples with edible films supplemented with oregano essential oil—OEOS ( **–** ).

Typical FTIR spectra from 4000 to 650 cm^−1^ collected from each group of ham slices at 12 °C and at the end of the shelf-life are presented in Figure 11, for samples with or without HPP treatment. As shown in Figure 11, major peaks are observed at 3650–3100 cm^–1^, due to the presence of water in the samples, at 1748 cm^−1^ due to esters from lipids and at 1650 cm^–1^ due to moisture and amide I bands of the proteins. The spectra collected were similar to those reported previously for ham in the spectral region from 1730 to 850 cm^–1^ [58]. Other minor intensity peaks are observed at 2925 (–CH_2_ asymmetric stretch), 2853 (aliphatic –CH_2_ groups of fatty acids), 1154 (CO–O–C asymmetric stretch, glycogen and nucleic acids), and 1040–1042 cm^−1^ (primary amines, C–N stretch, C–O stretch, polysaccharides) [59,60,61]. The spectral changes were more intense during the storage temperature at 12 °C and less intense, in the cases were HPP treatment was applied. 

The performance indices of the validation PLS regression models are presented in Table 1, for each group of samples (without or with HPP treatment) and for each bacterial group (LAB, lactococci/streptococci and TVC). The bias factor (B*_f_*) was estimated close to one for all the cases, showing good correlation between observations and predictions. On the other hand, the accuracy factor (A*_f_*) was estimated to be different between the cases with regard to HPP. Values close to one were observed for the samples without the HPP treatment, which shows that predictions were generally close to observations, while in the cases with HPP treatment, the values were quite higher. Specifically, the average deviation between predictions and observations were 8.2% for LAB, 6.6% for lactococci/streptococci and 5.9% for TVC for the non HPP treated samples, while the corresponding numbers for HPP samples were 30.5% for LAB, 31.1% for lactococci/streptococci and 23.5% for TVC. Analogous results were observed for the RMSE index, with low estimated values for the cases without the HPP treatment (lowest value observed for TVC: 0.56), but quite higher for the cases when HPP treatment was applied, with the highest value estimated for lactococci/streptococci (1.735). For the cases without HPP treatment, the percentage of prediction error was higher than the acceptable (≥70%) for lactococci/streptococci and TVC, while for LAB, was estimated as 66.38%. For the cases with HPP treatment the PE% were quite low; 30.72 for LAB, 37.57 for lactococci/streptococci and 47.54% for TVC. 

## 4. Discussion

Sliced ham is one of the most popular and widely consumed RTE meat products in the market. The production of ham normally entails a thermal or curing processing, before it is suitable for consumption. The application of HPP as an alternative method instead of the thermal pasteurization gained a lot of attention the past decade, especially to be used for meat products and ham. A variety of HPP products can be found in USA and Japan, as well as in Spain, which is a pioneer in high pressure processed meat [13]. There are many studies available in the literature with regard to the efficacy of the HPP application in meat products and the limitations observed for each product category, including ham [4,62,63,64,65,66,67,68,69,70]. 

HPP treatment is deemed to be efficient for pathogen inactivation, especially for *Listeria monocytogenes* in meat and meat products and the findings of this study support this claim. *Listeria* counts were reduced from 3.91 to 2.74 log CFU/g after HPP at 500 MPa for 2 min at an ambient temperature. Similar results were obtained by Stollewerk [71], investigating the effect of HPP in sliced cured ham inoculated with *Listeria monocytogenes* at a level of 20 CFU/g. In this study, the application of 600 MPa for 5 min at 13 °C caused a 1.1 log reduction to the initial pathogen level, which remained below detection limit during storage period at 4 °C for 112 days. Interestingly, in the control samples of the same study (samples without HPP treatment) *Listeria* did not exhibit any growth despite the optimum pH, which is in accordance with the findings of the present study. In another study of Jofre et al. [62], HPP at 600 MPa for 6 min at 31 °C, caused a reduction from 3.5 log CFU/g to < 10 CFU/g in cooked ham and dry cured ham, while during refrigeration *Listeria* although was present, remained below the detection limit. In the control samples of the same study, *Listeria* in cooked ham exhibited a mild growth; 1–2 log increase, while in dry cured ham remained in the same levels throughout the refrigerated storage. On the contrary, different observations were made by Koseki et al. [72], when HPP treatment did not eliminate the presence of *Listeria monocytogenes* in dry cured ham. Specifically, the use of HPP caused an initial reduction to *Listeria* levels from 5 log CFU/g to <10 CFU/g (detection limit), however, the pathogen showed a gradual growth and reached the levels of 7–8 log CFU/g after 70 days of storage at 10 °C. It has to be noted that HPP treatment below 450 MPa did not seem to affect *Listeria* counts and reduction of less than 1 log was observed, regardless of the treatment time and temperature [73].

To enhance the efficiency of HPP treatment on *Listeria monocytogenes* inactivation, other hurdles can also be applied, in combination with HPP, such as the presence of antimicrobials or active antimicrobial packaging, to enhance a pathogen-free product. Several studies are available dealing with the combined application of both technologies with generally promising results for pathogen inactivation in meat products [74,75,76,77,78]. In the present study, oregano essential oil was incorporated into Na-alginate edible films and its efficiency against *Listeria monocytogenes* and spoilage microbiota was examined with or without the application of HPP. When only HPP was used, an initial decrease of 1.2 log CFU/g in *Listeria* counts was observed, while the use of OEOS edible films led to a reduction of 1.5 logs in the end of storage period at 8 and 12 °C with the highest reduction observed at 4 °C (2.5 logs). The combination of HPP and OEOS films led to the reduction of *Listeria* counts below the detection limit and also in absence of the pathogen from almost the middle of the storage period. These results are similar with those previously reported by Jofre et al. [76], where HPP treatment of 600 MPa for 5 min was combined with the use of the antimicrobials nisin and potassium lactate to inactivate *Listeria monocytogenes* in sliced cooked ham. Consequently, HPP caused a 3 log reduction to *Listeria* counts, while with the use of the antimicrobials no growth was observed throughout the storage time at 1 and 6 °C (<10 CFU/g). In another study, the efficiency of enterocins as natural antimicrobials together with HPP against *Listeria monocytogenes* in sliced cooked ham was investigated. The authors used a pressure of 400 MPa for 10 min and the antimicrobials with a *Listeria* inoculum of 4.5–5.0 log CFU/g and storage temperatures of 1 and 6 °C. The combination of HPP, enterocins and storage at 1 °C led to a reduction of *Listeria* levels, while the use of enterocins was the most effective when combined with HPP compared to the use of lactate-diacetate and HPP [74]. 

In the present study, *Listeria* growth in ham samples was not observed in any case, including the control samples. A possible explanation can be the levels of the nitrates/nitrites present in the samples. It has to be noted that another critical factor for the growth of *Listeria* is the pH. The pH of the control samples without HPP, was found to be reduced faster compared to the control samples with HPP. The relatively fast drop of the pH, as a result of the increase of the population of the spoilage microbiota (LAB, lactococci/streptococci), could have also affected the fate of *Listeria*. Generally, the pH values for all the cases were decreased by time, while the decrease was less intense in the cases of HPP application. The storage temperature affected the final pH values, with the lowest ones to be observed at the storage temperature of 12 °C (abuse temperature). 

Supplementary in this study, PFGE was used in order to monitor the distribution or presence/absence of the different inoculated strains of *Listeria monocytogenes* in each tested case. Based on the results, it was evident that *Listeria* survival was a strain-dependent attribute. Differences were observed in the survival of each strain due to HPP and OEOS edible films application. Strain B133, which was isolated from soft cheese, was barely detected after the HPP application. Due to the fact that the other two strains (B129 and B131) were detected after HPP and where isolated from RTE food, similarly to the matrix used in the current study, it can be assumed that the source of isolation of each strain is important for the fate of *Listeria* when present in other ecological niches. However, it has to be noted that during the selective enrichment step, the strain competition of *Listeria* could have led to false-negative detection results [3]. 

The color values of the samples were determined in the study, due to their importance on quality assessment of the ham by the consumers. The color values were affected by the application of OEOS edible films (*p* < 0.05) and exhibited higher scores in all temperatures, while HPP treatment had no effect (*p* > 0.05) on the color. In a previous study [79], it was reported that the incorporation of oregano and thyme essential oils in inulin/chitosan blend films resulted in decreased L* (lightness) value and increased both a* (redness) and b* (yellowness) values of the films. In addition, in another study [80], the addition of carvacrol and cinnamaldeyde into carrot, apple and hibiscus-based edible films against *Listeria monocytogenes* in contaminated ham and bologna, showed differences in color compared to the control films. Different results, however, were observed in a study of Muriel-Galet et al. [40], when ethylene vinyl alcohol polymer (EVOH) supplemented with oregano essential oil at a 5% w/w dry polymer weight was used and no differences were noted in the color value of the EVOH film, after the addition of the essential oil. Regarding the effect of the HPP on the color of the sliced ham, many reports are available in the literature supporting that HPP can alter the color of the treated meat products, something that was not observed in our study [10,11,13]. The final color of the treated ham can be also affected by the fat, salt and water content of the product, as well as from the parameters involved in the HPP treatment (pressure applied and duration). 

The HPP treated samples were evaluated with overall better scores (lower) with regard to the sensorial characteristics compared to the samples without HPP treatment. It has to be highlighted that the HPP had no negative effect on the appearance of the ham slices. One of the disadvantages in the use of HPP is the potential effect of the pressure in the color and texture of the fresh products. Usually, HPP has low impact on thermal processed or cured products including ham, although the pressure values are considered critical for the extent of the effects [13]. For the case of the OEOS edible films, the aroma of the ham slices was assessed positively (lower scores/more fresh) and are considered as very promising for future applications. This observation was unexpected, due to the intense flavor of the OEO which could potentially have led to a rejection of these samples from the tasting panel. Pesavento [34], reported that essential oil concentrations more than 0.5% (v/w) in beef meatballs, resulted in very intense odor of the essential oil, while concentrations more that 2% (v/w) were deemed as unacceptable by the panelists. In the present study, the intensity of the essential oil flavor was masked by the presence of edible film, which mitigated the adverse effect of the oregano essential oil addition to the ham. In another study [81], the addition of OEO to poultry meat resulted in more acceptable aroma and flavor in comparison with samples without OEO addition. In addition, Qin [82], reported that chitosan with tea polyphenols enhanced the aroma and the acceptability of pork patties. 

FTIR spectroscopy revealed differences in samples treated and not treated with HPP and the estimated performance indices of the PLS-R models were found to be slightly different between the cases. A*_f_* values for the non-treated samples were close to one, indicating low average deviations between predictions and observations of the examined microbial groups, however the values of the same index were higher in the case of HPP. On the other hand, the estimated values for B*_f_* were in both cases very close to one, especially for the prediction of TVC, revealing good correlation between observations and predictions. Prediction error (%) values were found to be better for the cases without HPP treatment, with acceptable levels (>70%) for the prediction of lactococci/streptococci and TVC, while for the LAB the value was slightly lower than the acceptable level. Differently, in the samples treated with HPP, the PE values were quite low for all microbial groups, and thus not acceptable. Based on the results, it is evident that HPP treatment affected significantly the performance of the FTIR models to be used as a tool for the prediction of the actual microbial counts. Similar results were also observed in the previous study of our group, where probiotic-supplemented edible films were used instead of OEOS ones [54]. These results can be explained since HPP as a treatment could lead to changes in the matrix of the sliced ham, while ham during its production has received other additions or processes. Generally, it is worth to be mentioned that FTIR spectroscopy has been extensively used to monitor spoilage in raw meat [60,83,84,85,86], or to detect frozen-then-thawed meat [87], but applications and reports in food products such as ham are limited. 

## 5. Conclusions

The application of HPP and OEOS edible films, when used separately on ham slices affected the growth and survival of *Listeria monocytogenes* at 4, 8 and 12 °C. The combined application of the aforementioned treatments was found to be the most successful and resulted in a reduction of the pathogen’s counts in a shorter time with the lowest final levels. The HPP did not affect negatively the sensory characteristics of the ham slices, while the addition of OEOS edible films enhanced the aroma of the new products. The use of FTIR spectroscopy in tandem with chemometrics to assess the microbiological status and therefore the spoilage of the ham, was found to be adequate for some of the cases, however, revealed difficulties when HPP treatment was applied. The results of the study support the initial hypothesis that HPP and the addition of OEOS edible films in RTE meat products can eliminate the growth and even the presence of *Listeria monocytogenes*, and thus constitute an intriguing outcome for the food industry.

The HPP is a technology that could be utilized in the meat industry, as a second step, i.e., after the slicing stage of the ham, in order to eliminate spoilage and prevent the product from post-thermal cross-contamination. The selection of the ideal time-pressure combination is a critical parameter in a successful HPP application, and requires further customized investigation. Furthermore, the application of Na-alginate films supplemented with antimicrobials in sliced ham could be a good choice providing special organoleptic attributes to the product, whilst prolonging the shelf-life, due to the antimicrobial potential of these films. However, a possible difficulty in the use of edible films is the time required to prepare such films and the addition of the essential oils, even if added in small volumes. Considering these, such a technology would lead to an increase in the cost of the final product, which probably would affect the consumers’ options.

## Figures and Tables

**Table 1:** Performance indices of the partial least squares (PLS) regression (PLS-R) model, correlating the lactic acid bacteria (LAB), lactococci/streptococci and total viable counts (TVC) in each group of ham samples with regards to the high pressure processing (HPP) application, on the basis of FTIR spectral data.

Performance Indice	Without HPP Treatment			With HPP Treatment		
	LAB	Cocci	TVC	LAB	Cocci	TVC
Prediction error (PE, %)	66.38	74.29	77.40	30.72	37.57	47.54
Root mean square error (RMSE)	0.730	0.624	0.560	1.548	1.735	1.452
B*_f_*	0.973	0.987	0.988	0.942	0.961	1.001
A*_f_*	1.082	1.066	1.059	1.305	1.311	1.235

## References

[1] European Food Safety Authority and European Centre for Disease Prevention and Control (2018). The European Union summary report on trends and sources of zoonoses, zoonotic agents and food-borne outbreaks in 2017. EFSA J..

[2] Dussault D., Dang Vu K., Lacroix M. (2016). Developing of a model describing the inhibitory effect of selected preservatives on the growth of *Listeria monocytogenes* in a meat model system. Food Microbiol..

[3] Zilelidou E., Manthou E., Skandamis P. (2016). Growth differences and competition between *Listeria monocytogenes* strains determine their predominance on ham slices and lead to bias during selective enrichment with the ISO protocol. Int. J. Food Microbiol..

[4] Liu G., Wang Y., Gui M., Zheng H., Dai R., Li P. (2012). Combined effect of high hydrostatic pressure and enterocin LM-2 on the refrigerated shelf-life of ready-to-eat sliced vacuum-packed cooked ham. Food Control.

[5] Ramaroson M., Guillou S., Rossero A., Reze S., Anthoine V., Moriceau N., Martin J.-L., Duranton F., Zagorec M. (2018). Selection procedure of bioprotective cultures for their combined use with high pressure processing to control spore-forming bacteria in cooked ham. Int. J. Food Microbiol..

[6] Amaro-Blanco G., Delgado-Adámez J., Martín M.J., Ramírez R. (2018). Active packaging using an olive leaf extract and high pressure processing for the preservation of sliced-dry cured shoulders from Iberian pigs. Innov. Food Sci. Emerg. Technol..

[7] Odila Pereira J., Soares J., Monteiro M.J.P., Gomes A., Pintado M. (2018). Impact of whey protein coating incorporated with *Bifidobacterium* and *Lactobacillus* on sliced ham properties. Meat Sci..

[8] Varela-Santos E., Ochoa-Martinez A., Tabilo-Munizaga G., Reyes J.E., Pérez-Won M., Briones-Labarca V., Morales-Castro J. (2012). Effect of high hydrostatic pressure (HHP) processing on physicochemical properties, bioactive compounds and shelf-life of pomegranate juice. Innov. Food Sci. Emerg. Technol..

[9] Bak K.H., Lindahl G., Karlsson A.H., Lloret E., Ferrini G., Arnau J., Orlien V. (2012). High pressure effect on the color of minced cured restructured ham at different levels of drying, pH, and NaCl. Meat Sci..

[10] Hugas M., Garriga M., Monfort J.M. (2002). New mild technologies in meat processing: High pressure as a model technology. Meat Sci..

[11] Cheftel J.C., Culioli J. (1997). Effects of high pressure on meat: A review. Meat Sci..

[12] Torres J.A., Velazquez G. (2005). Commercial opportunities and research challenges in the high pressure processing of foods. J. Food Eng..

[13] Simonin H., Duranton F., de Lamballerie M. (2012). New insights into the high pressure processing of meat and meat products. Compr. Rev. Food Sci. Food Saf..

[14] McMillin K.W. (2017). Advancements in meat packaging. Meat Sci..

[15] (2009). Commission Regulation (EC) No 450/2009 of 29 May 2009 on active and intelligent materials and articles intended to come into contact with food. Off. J. Eur. Union.

[16] Kapetanakou A.E., Skandamis P.N. (2016). Applications of active packaging for increasing microbial stability in foods: Natural volatile antimicrobial compounds. Curr. Opin. Food Sci..

[17] Ribeiro-Santos R., Andrade M., Ramos de Melo N.R., Sanches-Silva A. (2017). Use of essential oils in active food packaging: Recent advances and future trends. Trends Food Sci. Technol..

[18] Kapetanakou A.E., Karyotis D., Skandamis P.N. (2016). Control of *Listeria monocytogenes* by applying ethanol-based antimicrobial edible films on ham slices and microwave-reheated frankfurters. Food Microbiol..

[19] Vermeiren L., Devlieghere F., van Beest M., de Kruijf N., Debevere J. (1999). Developments in active packaging of foods. Trends Food Sci. Technol..

[20] Coma V. (2008). Bioactive packaging technologies for extended shelf life of meat-based products. Meat Sci..

[21] De Azeredo H.M.C. (2013). Antimicrobial nanostructures in food packaging. Trends Food Sci. Technol..

[22] Ahmed I., Lin H., Zou L., Brody A.L., Li Z., Qazi I.M., Pavase T.R., Lv L. (2017). A comprehensive review on the application of active packaging technologies to muscle foods. Food Control.

[23] Wyrwa J., Barska A. (2017). Innovations in the food packaging market: Active packaging. Eur. Food Res. Technol..

[24] Zanetti M., Carniel T.K., Dalcanton F., dos Anjos R.S., Gracher Riella H., de Araújo P.H.H., de Oliveira D., Antônio Fiori M. (2018). Use of encapsulated natural compounds as antimicrobial additives in food packaging: A brief review. Trends Food Sci. Technol..

[25] Yildirim S., Röcker B., Pettersen M.K., Nilsen-Nygaard J., Ayhan Z., Rutkaite R., Radusin T., Suminska P., Marcos B., Coma V. (2018). Active packaging applications for food. Compr. Rev. Food Sci. Food Saf..

[26] Atares L., Chiralt A. (2016). Essential oils as additives in biodegradable films and coatings for active food packaging. Trends Food Sci. Technol..

[27] Skandamis P., Tsigarida E., Nychas G.-J.E. (2002). The effect of oregano essential oil on survival/death of *Salmonella typhimurium* in meat stored at 5 °C under aerobic, VP/MAP conditions. Food Microbiol..

[28] Deegan L.H., Cotter P.D., Hill C., Ross P. (2006). Bacteriocins: Biological tools for bio-preservation and shelf-life extension. Int. Dairy J..

[29] Kykkidou S., Giatrakou V., Papavergou A., Kontominas M.G., Savvaidis I.N. (2009). Effect of thyme essential oil and packaging treatments on fresh Mediterranean swordfish fillets during storage at 4 °C. Food Chem..

[30] Gutierrez J., Barry-Ryan C., Bourke P. (2009). Antimicrobial activity of plant essential oils using food model media: Efficacy, synergistic potential and interactions with food components. Food Microbiol..

[31] Jayasena D.D., Jo C. (2013). Essential oils as potential antimicrobial agents in meat and meat products. Trends Food Sci. Technol..

[32] Muriel-Galet V., Cerisuelo J.P., López-Carballo G., Aucejo S., Gavara R., Hernández-Muñoz P. (2013). Evaluation of EVOH-coated PP films with oregano essential oil and citral to improve the shelf-life of packaged salad. Food Control.

[33] Calo J.R., Crandall P.G., O’Bryan C.A., Ricke S.C. (2015). Essential oils as antimicrobials in food systems-A review. Food Control.

[34] Pesavento G., Calonico C., Bilia A.R., Barnabei M., Calesini F., Addona R., Mencarelli L., Carmagnini L., Di Martino M.C., Lo Nostro A. (2015). Antibacterial activity of Oregano, Rosmarinus and Thymus essential oil against *Staphylococcus aureus* and *Listeria monocytogenes* in beef meatballs. Food Control.

[35] Dimitrijević S.I., Mihajlovski K.R., Antonović D.G., Milanović-Stevanović M.R., Mijin D.Ž. (2007). A study of the synergistic antilisterial effects of a sub-lethal dose of lactic acid and essential oils from *Thymus vulgaris* L., *Rosmarinus officinalis*, L. and *Origanum vulgare*, L.. Food Chem..

[36] .Menezes N.M.C., Martins W.F., Longhi D.A., de Aragão G.M.F. (2018). Modelling the effect of oregano essential oil on shelf-life extension of vacuum-packed cooked sliced ham. Meat Sci..

[37] Tsigarida E., Skandamis P., Nychas G.-J.E. (2000). Behaviour of *Listeria monocytogenes* and autochthonous flora on meat stored under aerobic, vacuum and modified atmosphere packaging conditions with or without the presence of oregano essential oil at 5 °C. J. Appl. Microbiol..

[38] Dussault D., Vu K.D., Lacroix M. (2014). In vitro evaluation and antimicrobial activities of various commercial essential oils, oleoresin and pure compounds against food pathogens and application in ham. Meat Sci..

[39] Paparella A., Mazzarrino G., Chavez-López C., Rossi C., Sacchetti G., Guerrieri O., Serio A. (2016). Chitosan boosts the antimicrobial activity of *Origanum vulgare* essential oil in modified atmosphere packaged pork. Food Microbiol..

[40] Muriel-Galet V., Cran M.J., Bigger S.W., Hernández-Muñoz P., Gavara R. (2015). Antioxidant and antimicrobial properties of ethylene vinyl alcohol copolymer films based on the release of oregano essential oil and green tea extract components. J. Food Eng..

[41] Yanishlieva N.V., Marinova E.M., Gordon M.H., Raneva V.G. (1999). Antioxidant activity and mechanism of action of thymol and carvacrol in two lipid systems. Food Chem..

[42] Acosta S., Chiralt A., Santamarina P., Rosello J., González-Martínez C., Cháfer M. (2016). Antifungal films based on starch-gelatin blend, containing essential oils. Food Hydrocoll..

[43] Ruiz-Navajas Y., Viuda-Martos M., Sendra E., Perez-Alvarez J.A., Fernández-López J. (2013). In vitro antibacterial and antioxidant properties of chitosan edible films incorporated with *Thymus moroderi* or *Thymus piperella* essential oils. Food Control.

[44] Oussalah M., Caillet S., Salmiéri S., Saucier L., Lacroix M. (2007). Antimicrobial effects of alginate-based films containing essential oils on *Listeria monocytogenes* and *Salmonella typhimurium* present in bologna and ham. J. Food Protect..

[45] Pattanayaiying R., H-Kittikun A., Cutter C.N. (2015). Incorporation of nisin Z and lauric arginate into pullulan films to inhibit foodborne pathogens associated with fresh and ready-to-eat muscle foods. Int. J. Food Microbiol..

[46] Costa M.J., Maciel L.C., Teixeira J.A., Vicente A.A., Cerqueira M.A. (2018). Use of edible films and coatings in cheese preservation: Opportunities and challenges. Food Res. Int..

[47] Varela P., Fiszman S.M. (2011). Hydrocolloids in fried foods. A review. Food Hydrocoll..

[48] Panagou E.Z., Tassou C.C., Manitsa C., Mallidis C. (2007). Modelling the effect of high pressure on the inactivation kinetics of a pressure-resistant strain of *Pediococcus damnosus* in phosphate buffer and gilt-head seabream (*Sparus aurata*). J. Appl. Microbiol..

[49] Tassou C.C., Panagou E.Z., Samaras F.J., Galiatsatou P., Mallidis C.G. (2008). Temperature-assisted high hydrostatic pressure inactivation of *Staphylococcus aureus* in a ham model system: Evaluation in selective and nonselective medium. J. Appl. Microbiol..

[50] International Organization for Standardization (ISO) (2004). Microbiology of Food and Animal Feeding Stuffs—Horizontal Method for the Detection and Enumeration of Listeria monocytogenes. Part 1: Detection Method.

[51] Kagkli D.M., Iliopoulos V., Stergiou V., Lazaridou A., Nychas G.-J.E. (2009). Differential *Listeria monocytogenes* strain survival and growth in Katiki, a traditional Greek soft cheese, at different storage temperatures. Appl. Environ. Microbiol..

[52] Pavli F., Kovaiou I., Apostolakopoulou G., Kapetanakou A., Skandamis P., Nychas G.-J.E., Tassou C., Chorianopoulos N. (2017). Alginate-Based edible films delivering probiotic bacteria to sliced ham pretreated with high pressure processing. Int. J. Mol. Sci..

[53] Gill C.O., Jeremiah L.E. (1991). The storage life of non-muscle offals packaged under vacuum or carbon dioxide. Food Microbiol..

[54] Pavli F., Argyri A.A., Nychas G.-J.E., Tassou C., Chorianopoulos N. (2018). Use of Fourier transform infrared spectroscopy for monitoring the shelf life of ham slices packed with probiotic supplemented edible films after treatment with high pressure processing. Food Res. Int..

[55] Ross T. (1996). Indices for performance evaluation of predictive models in food microbiology. J. Appl. Bacteriol..

[56] Oscar T.P. (2009). Predictive model for survival and growth of *Salmonella typhimurium* DT104 on chicken skin during temperature abuse. J. Food Protect..

[57] Oscar T.P. (2005). Validation of lag time and growth rate models for *Salmonella* Typhimurium: Acceptable prediction zone method. J. Food Sci..

[58] Moreirinha C., Nunes A., Barros A., Almeida A., Delgadillo I. (2015). Evaluation of the potential of mid-infrared spectroscopy to assess the microbiological quality of ham. J. Food Saf..

[59] Böcker U., Ofstad R., Wu Z., Bertram H.C., Sockalingum G.D., Manfait M., Egelandsdal B., Kohler A. (2007). Revealing covariance structures in Fourier transform infrared and Raman microspectroscopy spectra: A study on pork muscle fiber tissue subjected to different processing parameters. Appl. Spectrosc..

[60] Ammor S.A., Argyri A., Nychas G.J.E. (2009). Rapid monitoring of the spoilage of minced beef stored under conventionally and active packaging conditions using Fourier transform infrared spectroscopy in tandem with chemometrics. Meat Sci..

[61] Osorio M.T., Zumalacarregui J.M., Alaiz-Rodriguez R., Gusman-Martinez R., Englesen S.B., Mateo J. (2009). Differentiation of perirenal and omental quality of suckling lamps according to the rearing system from Fourier transforms mid-infrared spectra using partial least squares and artificial neural networks analysis. Meat Sci..

[62] Jofré A., Aymerich T., Grèbol N., Garriga M. (2009). Efficiency of high hydrostatic pressure at 600 MPa against food-borne microorganisms by challenge tests on convenience meat products. LWT Food Sci. Technol..

[63] Han Y., Jiang Y., Xu X., Sun X., Xu B., Zhou G. (2011). Effect of high pressure treatment on microbial populations of sliced vacuum-packed cooked ham. Meat Sci..

[64] Vercammen A., Vanoirbeek K.G.A., Lurquin I., Steen L., Goemaere O., Szczepaniak S., Paelinck H., Hendrickx M.E.G., Michiels C.W. (2011). Shelf-Life extension of cooked ham model product by high hydrostatic pressure and natural preservatives. Innov. Food Sci. Emerg. Technol..

[65] Myers K., Montoya D., Cannon J., Dickson J., Sebranek J. (2013). The effect of high hydrostatic pressure, sodium nitrite, and salt concentration of the growth of *Listeria monocytogenes* on RTE ham and turkey. Meat Sci..

[66] Belletti N., Garriga M., Aymerich T., Bover-Cid S. (2013). Inactivation of *Serratia liquefaciens* on dry-cured ham by high pressure processing. Food Microbiol..

[67] Hereu A., Dalgaard P., Garriga M., Aymerich T., Bover-Cid S. (2014). Analysing and modelling the growth behavior of *Listeria monocytogenes* on RTE cooked meat products after a high pressure treatment at 400 MPa. Int. J. Food Microbiol..

[68] Pietrasik Z., Gaudette N.J., Johnston S.P. (2016). The use of high pressure processing to enhance the quality and shelf life of reduced sodium naturally cured restructured cooked hams. Meat Sci..

[69] Pingen S., Sudhaus N., Becker A., Krischek C., Klein G. (2016). High pressure as an alternative processing step for ham production. Meat Sci..

[70] Rubio B., Possas A., Rincón F., García-Gimeno R.M., Martínez B. (2018). Model for *Listeria monocytogenes* inactivation by high hydrostatic pressure processing in Spanish chorizo sausage. Food Microbiol..

[71] Stollewerk K., Jofré A., Comaposada J., Arnau J., Garriga M. (2012). The effect of NaCl-free processing and high pressure on the fate of *Listeria monocytogenes* and *Salmonella* on sliced smoked dry-cured ham. Meat Sci..

[72] Koseki S., Mizuno Y., Yamamoto K. (2007). Predictive modelling of the recovery of *Listeria monocytogenes* on sliced cooked ham after high pressure processing. Int. J. Food Microbiol..

[73] Bover-Cid S., Belletti N., Garriga M., Aymerich T. (2011). Model for *Listeria monocytogenes* inactivation on dry-cured ham by high hydrostatic pressure processing. Food Microbiol..

[74] Marcos B., Jofrè A., Aymerich T., Monfort J.M., Garriga M. (2008). Combined effect of natural antimicrobials and high pressure processing to prevent *Listeria monocytogenes* growth after a cold chain break during storage of cooked ham. Food Control.

[75] Marcos B., Aymerich T., Monfort J.M., Garriga M. (2008). High-pressure processing and antimicrobial biodegradable packaging to control *Listeria monocytogenes* during storage of cooked ham. Food Microbiol..

[76] Jofré A., Garriga M., Aymerich T. (2008). Inhibition of *Salmonella* sp., *Listeria monocytogenes* and *Staphylococcus aureus* in cooked ham by combining antimicrobials, high hydrostatic pressure and refrigeration. Meat Sci..

[77] Stratakos A.C., Delgado-Pando G., Linton M., Patterson M.F., Koidis A. (2014). Synergism between high-pressure processing and active packaging against *Listeria monocytogenes* in ready-to-eat chicken breast. Innov. Food Sci. Emerg. Technol..

[78] Teixeira J.S., Repková L., Gänzle M.G., McMullen L.N. (2018). Effect of pressure, reconstituted RTE meat microbiota, and antimicrobials on survival and post-pressure growth of *Listeria monocytogenes* on ham. Front. Microbiol..

[79] Cao T.L., Yang S.Y., Song K.B. (2018). Development of burdock root inulin/chitosan blend films containing oregano and thyme essential oils. Int. J. Mol. Sci..

[80] Ravishankar S., Jaroni D., Zhu L., Olsen C., McHugh T., Friedman M. (2012). Inactivation of *Listeria monocytogenes* on ham and bologna using pectin-based apple, carrot, and hibiscus edible films containing carvacrol and cinnamaldehyde. J. Food Sci..

[81] Petrou S., Tsiraki M., Giatrakou V., Savvaidis I.N. (2012). Chitosan dipping or oregano oil treatments, singly or combined on modified atmosphere packaged chicken breast meat. Int. J. Food Microbiol..

[82] Qin Y.-Y., Jang J.-Y., Lu H.-B., Wang S.-S., Yang J., Yang X.-C., Chai M., Li L., Cao J.-X. (2013). Effect of chitosan film incorporated with tea polyphenol on quality and shelf life of pork meat patties. Int. J. Biol. Macromol..

[83] Papadopoulou O., Panagou E.Z., Tassou C.C., Nychas G.J.E. (2011). Contribution of Fourier transform infrared (FTIR) spectroscopy data on the quantitative determination of minced pork meat spoilage. Food Res. Int..

[84] Panagou E.Z., Mohareb F.R., Argyri A.A., Bessa C.M., Nychas G.J.E. (2011). A comparison of artificial neural networks and partial least squares modeling for the rapid detection of the microbial spoilage of beef fillets based on Fourier transform infrared spectral fingerprints. Food Microbiol..

[85] Argyri A.A., Jarvis R.M., Wedge D., Xu Y., Panagou E.Z., Goodacre R., Nychas G.-J.E. (2013). A comparison of Raman and FT-IR spectroscopy for the prediction of meat spoilage. Food Control..

[86] Estelles-Lopez L., Ropodi A., Pavlidis D., Fotopoulou J., Gkousari C., Peyrodie A., Panagou E., Nychas G.-J.E., Mohareb F. (2017). An automated ranking platform for machine learning regression models for meat spoilage prediction using multi-spectral imaging and metabolic profiling. Food Res. Int..

[87] Ropodi A.I., Panagou E.Z., Nychas G.-J.E. (2018). Rapid detection of frozen-then-thawed minced beef using multispectral imaging and Fourier transform infrared spectroscopy. Meat Sci..

